# Complement Activation in Placental Malaria

**DOI:** 10.3389/fmicb.2015.01460

**Published:** 2015-12-21

**Authors:** Chloe R. McDonald, Vanessa Tran, Kevin C. Kain

**Affiliations:** ^1^Sandra Rotman Laboratories, Sandra Rotman Centre for Global Health, Toronto General Research Institute, University Health Network, TorontoON, Canada; ^2^Department of Global Health and Population, Harvard School of Public Health, BostonMA, USA; ^3^Tropical Disease Unit, Division of Infectious Diseases, Department of Medicine, University of Toronto, TorontoON, Canada

**Keywords:** malaria, pregnancy, placental malaria, complement, inflammation, angiogenesis, neurodevelopment

## Abstract

Sixty percent of all pregnancies worldwide occur in malaria endemic regions. Pregnant women are at greater risk of malaria infection than their non-pregnant counterparts and have a higher risk of adverse birth outcomes including low birth weight resulting from intrauterine growth restriction and/or preterm birth. The complement system plays an essential role in placental and fetal development as well as the host innate immune response to malaria infection. Excessive or dysregulated complement activation has been associated with the pathobiology of severe malaria and with poor pregnancy outcomes, dependent and independent of infection. Here we review the role of complement in malaria and pregnancy and discuss its part in mediating altered placental angiogenesis, malaria-induced adverse birth outcomes, and disruptions to the *in utero* environment with possible consequences on fetal neurodevelopment. A detailed understanding of the mechanisms underlying adverse birth outcomes, and the impact of maternal malaria infection on fetal neurodevelopment, may lead to biomarkers to identify at-risk pregnancies and novel therapeutic interventions to prevent these complications.

## The Global Burden of Malaria

Nearly half of the world’s population remains at risk of malaria infection (WHO, 2013). Malaria is caused by the protozoan parasite *Plasmodium* and includes five species that infect humans: *Plasmodium falciparum, P. vivax, P. ovale, P. malaria, and P. knowlesi.* Among these, *P. falciparum* causes the most severe disease and accounts for the majority of malaria-associated deaths (Dellicour et al., 2010). Pregnant women are particularly susceptible to malaria-associated morbidity and mortality with approximately 125 million pregnancies at risk of infection each year (Dellicour et al., 2010). Malaria during pregnancy can result in anemia, stillbirth, and low birth weight (LBW) resulting from intrauterine growth restriction (IUGR) and/or preterm birth (PTB; Rogerson et al., 2003; Umbers et al., 2011; Eisele et al., 2012). These outcomes are associated with an increased risk of neonatal mortality and contribute to an estimated 200 000 infant deaths annually (Steketee et al., 2001; van Geertruyden et al., 2004). PTB, IUGR, and LBW have consistently been associated with developmental delay and an increased risk of long-term health consequences including cardiovascular disease, diabetes, and obesity (March of Dimes, PMNCH, Save the Children, WHO, 2012; Visentin et al., 2014). Further, a growing body of evidence has linked *in utero* exposure to infections to long-term cognitive and behavioral disorders including autism, schizophrenia, and depression (Knuesel et al., 2014). Despite the connection between prenatal infections and adverse neurological outcomes for the developing child, the potential impact of *in utero* exposure to malaria on subsequent neurodevelopment remains understudied.

## Pathophysiology of Placental Malaria

*Plasmodium falciparum* infection during pregnancy can result in placental malaria (PM), defined by the accumulation of parasitized erythrocytes (PEs) in the placental intervillous space and the infiltration of maternal monocytes/macrophages (Rogerson et al., 2003). The PEs that sequester in the placenta bind via a unique *P. falciparum* erythrocyte membrane protein 1 (PfEMP1) variant, VAR2CSA, to the glycosaminoglycan chondroitin sulfate A (CSA) that is expressed on the syncytiotrophoblast lining of the intervillous space (Duffy et al., 2006; Mens et al., 2010; Clausen et al., 2012). As such, protective immunity developed during exposure to malaria in non-pregnancy is ineffective such that primigravidae are at highest risk of PM and its associated poor birth outcomes (Desai et al., 2007). Adaptive immunity is gradually acquired during malaria infections in pregnancy and is mediated by the acquisition of anti-VAR2CSA adhesion blocking and opsonic antibodies (Fried et al., 1998; Desai et al., 2007; Keen et al., 2007).

Sequestration of PEs stimulates maternal macrophages to express β-chemokines, including monocyte chemotactic protein-1 (MCP-1), macrophage inflammatory protein (MIP)-1α, and MIP-1β, that recruit other inflammatory mediators and initiate the inflammatory cascade (Suguitan et al., 2003). This localized placental immune response and inflammation is thought to contribute to the adverse birth outcomes associated with PM. Although the precise mechanisms of placental and fetal injury are unclear, evidence suggests that the complement system may play a role.

## The Complement System

The complement system is a crucial immune surveillance and innate defense pathway. It is composed of both soluble and membrane bound proteins that cooperate to function in host defense and inflammation. Normally, the complement system is maintained at a basal level of activation but can be further amplified through three major activation pathways: the classical pathway, the mannose-binding lectin (MBL) pathway, and the alternative pathway (Ricklin et al., 2010; Wagner and Frank, 2010; Woodruff et al., 2011). The classical pathway is activated by binding of C1q to IgM or IgG immune complexes, the mannose-binding lectin pathway is activated by binding of foreign carbohydrate moieties, and the alternative pathway is activated by bacterial lipopolysaccharide (LPS) and negatively charged viral surfaces. The three pathways converge in a sequential cleavage cascade that results in opsonization-mediated phagocytosis, cell lysis, or an inflammatory response through the activation of the C3-convertase, which catalyzes the cleavage of C3 to C3a and C3b. C3b is an opsonizing fragment that binds to foreign antigens and increases phagocytosis. In addition, C3b can combine with C3-convertases to form the C5-convertase which cleaves C5 to C5a and C5b. C3a and C5a are potent anaphylatoxins that activate neutrophils and macrophages to promote inflammation. C5b recruits C6–C9 and forms the membrane attack complex (MAC), which can insert in cell membranes and lyse target cells. In addition to these traditional pathways, direct C3 and C5 cleavage can occur via thrombin or serine proteases (Huber-Lang et al., 2002; Ward, 2004; Huber-Lang et al., 2006).

The complement system is also an important regulator of several developmental processes, and as such requires tight regulation to prevent excessive activation (Ricklin and Lambris, 2007; Silver et al., 2010). Regulation is controlled through the expression of complement regulatory proteins including the complement receptor 1 (CR1), decay accelerating factor (DAF), and Factor H. These proteins control complement activation by binding effector proteins to limit activation or to accelerate degradation of complement components (Ricklin et al., 2010). The important role of complement in normal developmental processes is highlighted by several diseases that are linked to mutations in complement effector and regulatory proteins including, systemic lupus erythematosus, age-related macular degeneration, atypical hemolytic uremic syndrome (aHUS), paroxysmal nocturnal hemoglobinuria (PNH), and deficits in vascular development and metabolism (Seelen et al., 2003; Edwards et al., 2005; Haines et al., 2005; Klein et al., 2005; Risitano, 2012; Cofiell et al., 2015; Martinez-Barricarte et al., 2015).

## Complement Activation in Pregnancy and Obstetric Complications

The complement system plays an essential role in healthy pregnancies (Regal et al., 2015) protecting against local infection and regulating the maternal immune response to the semi-allogenic fetal tissue (Richani et al., 2005; Derzsy et al., 2010). During early pregnancy the trophoblast layer invades the decidua resulting in an inflammatory response controlled by trophoblast-derived complement regulatory proteins including membrane cofactor protein (MCP, CD46), protectin (CD59), and DAF (CD55; Tedesco et al., 1993). The placenta synthesizes complement components, providing a local source of protection from infection. Histological studies have reported positive staining for C1q, C9, C3d, C4BP, and Factor H in placentas from healthy term pregnancies. Human trophoblast cells secrete C3 and C4 proteins and phagocytic activity in the trophoblast is mediated by activated C3 in mouse trophoblast cells (Albieri et al., 1999; Bulla et al., 2009). Endothelial cells in the placenta secrete C1q, which appears at sites of trophoblast invasion into the decidual tissue (Bulla et al., 2008). Throughout pregnancy regulatory proteins produced by the chorion facilitate complement activity and prevent complement-mediated placental injury.

Excessive or dysregulated activation of the complement system can overwhelm these regulatory and protective pathways. Multiple studies have reported an association between complement split products (e.g., C3a and C5a) or mutations in complement regulatory proteins and obstetric complications (Tedesco et al., 1993; Lynch et al., 2011; Denny et al., 2013). An estimated 8–18% of women with preeclampsia have mutations in complement regulatory proteins (Salmon et al., 2011). Increased circulating levels of C5a, activated Factor B (Bb), C5b-9 and C3a/C3 ratio in pregnancy have been linked to spontaneous abortion, preeclampsia, hemolysis, and the elevated liver enzymes, low platelet count (HELLP) syndrome (Girardi et al., 2006a; Derzsy et al., 2010; Lynch and Salmon, 2010). Experimental models have provided mechanistic evidence that blockade of C5a, C5a receptor (C5aR), Factor B, C3, or C6 in non-infection models protects against fetal lethality and IUGR (Holers et al., 2002; Girardi et al., 2006b; Lynch et al., 2011; Singh et al., 2011). C5a may contribute to multiple complications in pregnancy via its ability to synergistically induce the secretion of pro-inflammatory (e.g., TNF, IL-8, IL-1β) and anti-angiogenic factors (e.g., soluble fms-like tyrosine kinase-1 [sFlt-1]) from leukocyte populations (Conroy et al., 2009; Langer et al., 2010). Cleavage of C5 has also been reported to induce monocyte recruitment, matrix metalloprotease production, and cervical ripening in a mouse model of LPS-induced PTB (Gonzalez et al., 2011, 2013). In this model, blocking C5a-C5aR signaling rescued offspring from cortical fetal brain injury (Pedroni et al., 2014). Finally, sC5b-9 in urine has been associated with an anti-angiogenic profile including elevated sFlt-1, and reduced placental growth factor (PGF) and vascular endothelial growth factor (VEGF; Guseh et al., 2015).

## Complement in Placental Malaria

**FIGURE 1:**
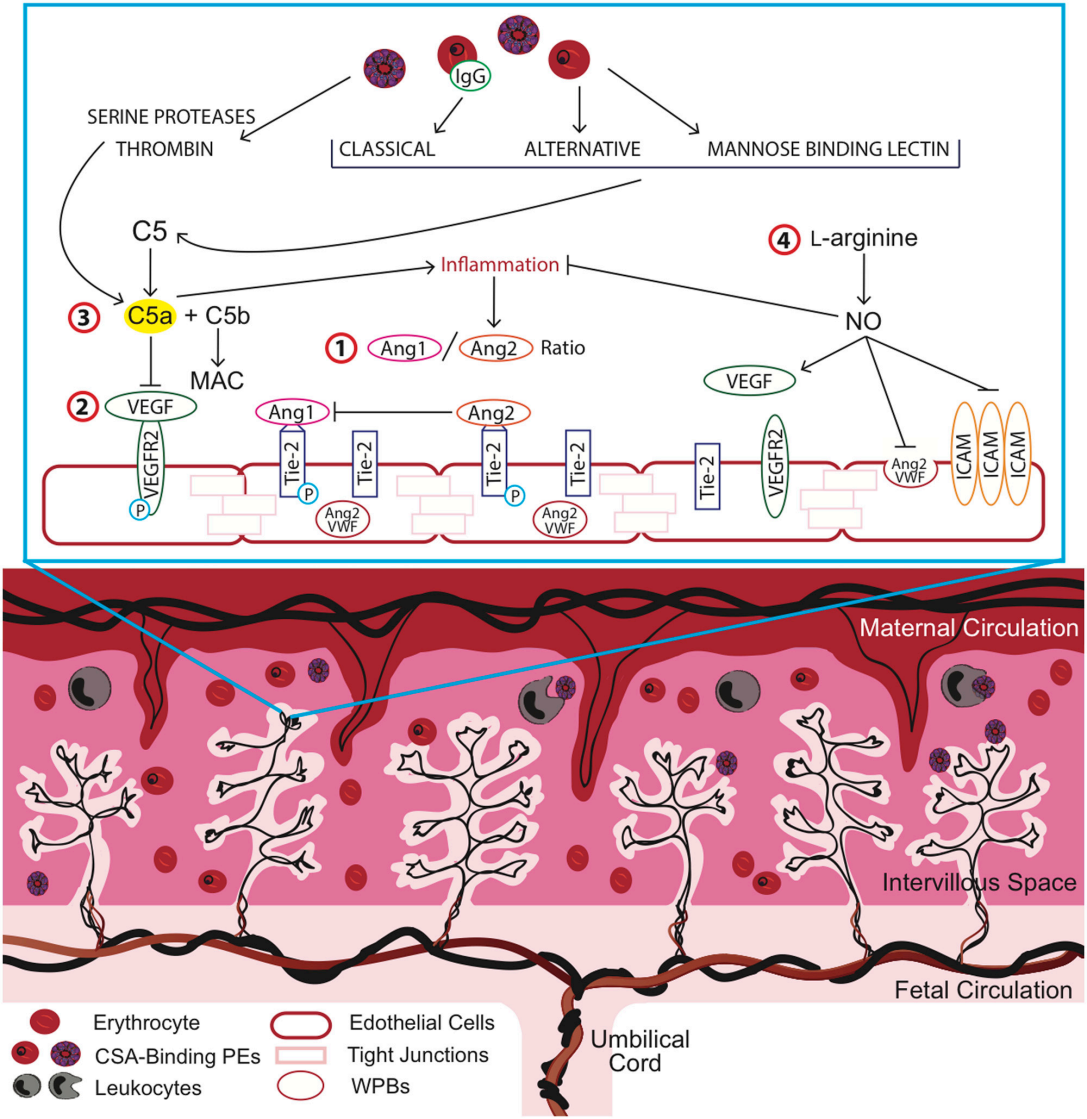
**Overview of key pathways in placental malaria (PM) and potential targets for therapeutic intervention to improve birth outcomes.** Strategies to optimize placental vascular development and function may impact the length of gestation, birth weight, and birth outcome by targeting complement and complement-associated pathways. Inflammation and dysregulation of angiogenic pathways has been observed in association with chondroitin sulfate A (CSA)-binding parasitized erythrocytes (PEs) in the intervillous space of the placenta. Key angiogenic factors such as **(1)** the angiopoietins (Ang), Ang-1 and Ang-2, which act as antagonists at the Tie-2 receptor and **(2)** vascular endothelial growth factor (VEGF), mediate vasculogenic and angiogenic processes in the placenta. **(3)** Malaria-induced complement activation resulting in the cleavage of C5a, a potent anaphylatoxin, contributes to increased inflammation and dysregulated angiogenesis and can be targeted via anti-C5a antibodies or alternatively, by more cost-effective strategies such as **(4)** L-arginine supplementation. L-arginine supplementation could increase bioavailable nitric oxide (NO), which acts to reduce malaria-induced inflammation at the maternal-fetal interface and promote vascular development. ICAM, intercellular adhesion molecule; MAC, membrane attack complex; VWF, von Willebrand factor.

Complement is a key component of the innate immune response to malaria and contributes to both protection (e.g., PE opsonization) and pathologic host responses (e.g., coagulopathy, inflammation, and endothelial activation; Silver et al., 2010; Biryukov and Stoute, 2014). Activation can occur by both traditional and non-traditional pathways (**Figure 1**). For example, IgG has been shown to be deposited on the surface of PEs and C-reactive protein has been shown to be elevated in malaria infection (Mibei et al., 2005), both of which can activate the classical pathway. Activation of the alternative pathway can occur from parasite-induced alterations to the erythrocyte surface that induce spontaneous C3 activation or by platelet-derived factor D following vascular damage (Roestenberg et al., 2007). Evidence for MBL pathway activation is inferred from a higher prevalence of MBL polymorphisms (e.g., MBL2.LXPA haplotype) in women with PM, however, no association was observed between levels of MBL and infection or birth outcomes (Thevenon et al., 2009). Finally, malaria can activate complement through C3-independent pathways through cleavage of C3 and C5 via thrombin and serine proteases released by phagocytic leukocytes or the parasite (Huber-Lang et al., 2002, 2006; Conroy et al., 2009).

This excessive complement activation during malaria infection can perturb the tight regulation that is important during a healthy pregnancy. Research in human populations and mouse models support the hypothesis that malaria-induced complement activation contributes to adverse birth outcomes by increasing inflammation and dysregulating angiogenic processes essential for normal placental development and function (Conroy et al., 2011, 2013). Several observational studies have reported elevated C5a in peripheral and placental blood in women with PM and linked these changes to adverse birth outcomes, including IUGR, PTB, and LBW (Conroy et al., 2011). Further, this was associated with dysregulated angiogenesis in the placenta (Conroy et al., 2009, 2013). Placental and fetal vascular development is regulated by several pathways, most notably the VEGF and Angiopoietin-Tie2 pathways (Geva et al., 2002; Charnock-Jones et al., 2004; Burton et al., 2009). The VEGF axis is essential for placental vascularization, vessel growth, and remodeling (Kingdom et al., 2000; Charnock-Jones et al., 2004; Kaufmann et al., 2004). VEGF and other pro-angiogenic factors in this family, such as PGF, interact with anti-angiogenic mediators (e.g., sFlt-1) to regulate endothelial cell survival, proliferation, migration, and sprouting (Demir et al., 2004, 2006). Malaria-induced complement activation may result in a shift to an anti-angiogenic profile (i.e., increased sFlt-1 and decreased PGF and VEGF) that can impede normal vasculogenic and angiogenic processes (Silver et al., 2011; Conroy et al., 2013). *In vitro*, C5a, in combination with parasite products, results in the synergistic release of pro-inflammatory mediators and anti-angiogenic factors, including sFlt-1, from human monocytes (Conroy et al., 2009). Complement-induced anti-angiogenic activity can disrupt these closely regulated processes and provoke changes in the placental vasculature. Genetic and pharmacological blockade of C5a-C5aR increases placental vascular length and segment number, reduces vascular resistance in the placenta, and increases fetal weight and viability (Conroy et al., 2013). This rescue is thought to be mediated in part by reducing the impact of C5a on the Ang/Tie2 pathway.

The angiopoietins, Ang-1 and Ang-2, are angiogenic factors that act in a context-dependent manner with VEGF to regulate placental vasculogenesis, angiogenesis, and vascular inflammation. The angiopoietins competitively bind to the Tie-2 receptor and act as antagonists to one another with Ang-1 inducing vascular maturation and Ang-2 causing destabilization of the vascular network and angiogenesis (Charnock-Jones et al., 2004). Decreased levels of circulating Ang-1 and elevated levels of Ang-2 were observed in malaria-infected pregnant women in Cameroon and Malawi (Silver et al., 2011; Conroy et al., 2013). Further, an increase in the ratio of Ang-2/Ang-1 was observed in Cameroonian women who delivered LBW infants and this effect was recapitulated in the mouse model of PM (Silver et al., 2010). These findings support the role of dysregulated angiogenesis in adverse birth outcomes associated with PM and highlight the potential for Ang/Tie2-targeted therapies as intervention strategies.

## Potential Therapeutic Strategies Targeting Complement and Associated Pathways

Optimizing placental vascular development and function and resulting changes in the *in utero* environment can impact the length of gestation, birth weight, and birth outcome. PTB is now the leading direct cause of infant mortality and the second leading cause of death in children under the age of five, after pneumonia (Blencowe et al., 2012; March of Dimes, PMNCH, Save the Children, WHO, 2012). Maternal infections are considered the major modifiable factor contributing to PTB, with the highest burden occurring in low resource settings. Despite the enormous public health impact of malaria in pregnancy, there are few effective interventions, and even fewer aimed at promoting healthy placental development. The above pathways highlight key angiogenic mediators (e.g., Ang/Tie2 and VEGF) altered during PM that can be investigated as targets for new interventions to improve birth outcomes in high-risk pregnancies (**Figure 1**).

Angiogenic-modifying compounds are currently being investigated therapeutically for a number of diseases, including anti-angiogenic therapies for metastatic cancer, wound healing, and pathological vasculopathies and retinopathies (Huang et al., 2010; Wietecha and DiPietro, 2013; Gacche and Meshram, 2014). Historically, most approaches have targeted VEGF to either restrict (anti-VEGF therapy as cancer therapeutics) or promote (VEGF therapy in wound healing) angiogenesis (Delli Carpini et al., 2010; Wietecha and DiPietro, 2013). Although early clinical trials yielded promising results in tumor stabilization and improved patient survival, resistance to VEGF-targeted therapies is emerging (Bergers and Hanahan, 2008; Ebos et al., 2009). Only recently has the Ang/Tie2 pathway been targeted for regulating angiogenesis. In cancer therapies, Tie2 inhibitors and Ang-1/-2 traps have been investigated and have shown promising anti-tumor growth activity in various cancer cell lines and good tolerability in early human trials (Huang et al., 2010).

Ang/Tie-2-targeted therapeutics have not been investigated in the context of PM, however, *in vivo* data using the mouse model of cerebral malaria have shown potential utility (Serghides et al., 2014). While this has yet to be evaluated in humans, it provides proof-of-principle evidence to support the use of Ang-targeted therapies. Given the observed decrease in circulating maternal Ang-1 and the associated dysregulated placental angiogenesis, therapies that regulate the Ang/Tie2 pathway may have clinical use in PM but there are important safety barriers to overcome with any of these agents.

A humanized monoclonal antibody to complement C5, Eculizumab, is approved to treat aHUS and PNH (Risitano, 2012). aHUS shares many pathophysiological features with malaria including release of free heme, complement activation, and endothelial dysfunction in association with the release of vWF and Ang-2 (Noris et al., 2014). PNH is caused by the reduced expression of complement regulatory proteins on hematopoietic cells resulting in intravascular hemolysis, thrombophilia, and cytopenias. During pregnancy, patients with PNH are at a greater risk of mortality (Ray et al., 2000). Case reports of Eculizumab for PNH during pregnancy suggest that blockade of C5 signaling does not negatively impact birth outcomes (Kelly et al., 2010; Marasca et al., 2010; Patriquin and Leber, 2015). Given that complement is an upstream regulator of angiogenesis and the trials of anti-C5 antibody in pregnancy report good tolerability, the use of anti-complement strategies to treat PM is promising (**Figure 1**). However, the high cost of anti-complement antibodies would limit its use in resource-constrained settings and therefore more affordable strategies need to be explored. For example, L-arginine is an essential amino acid in pregnancy and immediate precursor of nitric oxide (NO), an endogenous regulator of placental vascular development (Goodrum et al., 2003; Krause et al., 2011). There is substantial evidence that malaria-infection depletes L-arginine and greatly reduces bioavailable NO and that this contributes to the pathobiology of severe malaria (Anstey et al., 1996; Lopansri et al., 2003; Serghides et al., 2011; Weinberg et al., 2014). Low dietary intake of L-arginine is common in resource-constrained settings and further depletes bioavailable NO. L-arginine supplementation in pregnancy may promote placental vascular development and function by restoring bioavailable NO (**Figure 1**). Intervention strategies targeting the L-arginine-NO pathway are being discussed as potential therapeutics for preeclampsia and appear promising due to simple routes of administration, low cost, and a very low toxicity profile in pregnancy (Cindrova-Davies, 2014; Johal et al., 2014). Given the number of pregnancies at risk of malaria infection, in combination with the rising rates of PTB particularly in low resource settings, L-arginine supplementation may present a safe, affordable, and feasible intervention to reduce malaria immunopathology and protect the *in utero* environment.

## The Impact of Malaria on Neurocognitive Development of Offspring

**FIGURE 2:**
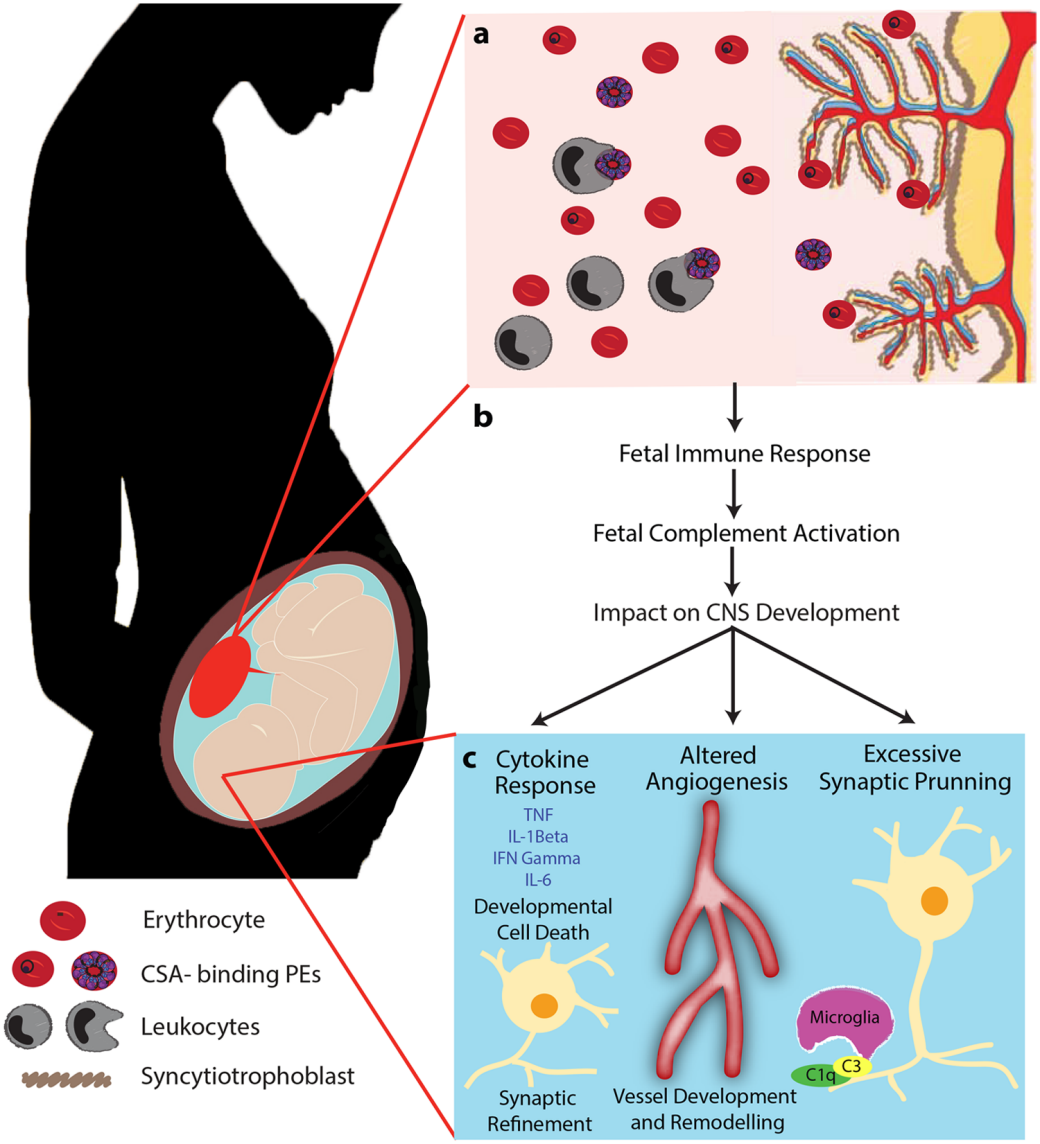
**Proposed mechanism of PM-mediated adverse birth outcomes and altered neurodevelopmental processes.** PM is characterized by sequestration of PEs in the intervillous space of the placenta resulting in the recruitment of leukocytes (monocytes and macrophages), complement activation, and localized inflammation **(A)**. We propose that this localized maternal inflammation induces an inflammatory and complement response in the fetus **(B)**. Subsequently, induced complement activation and dysregulation of complement effector and regulatory proteins impact neurodevelopmental processes by altering brain cytokine levels, dysregulating angiogenic processes, and altering normal synaptic pruning **(C)**. Initiation of the complement cascade induces maternal inflammation which can result in the presence of inflammatory mediators in the fetal brain. Complement may also impact fetal cerebral vascular development and dependent neurodevelopmental pathways. Finally, increased levels of complement can lead to excessive elimination of synapses and disrupt refinement of precise neural circuits that are critical to normal fetal neurodevelopment. Adapted from McDonald et al. (2013) Trends in Parasitology 29(5) 213–219. Adapted with permission.

It is well established that normal placental development is fundamental to optimal fetal growth. In addition to PM-induced changes in vascular development, malaria infection may interfere with complement-mediated pathways that regulate neurodevelopment *in utero* (**Figure 2**). Research has highlighted the pivotal role that the prenatal environment plays in postnatal neurodevelopment as well as later life cognition and behavior (Bilbo and Schwarz, 2009; Bale et al., 2010; Mwaniki et al., 2012). The impact of prenatal infection on neurodevelopmental processes has been examined in animal models and in children (Deverman and Patterson, 2009; Khandaker et al., 2012). Maternal viral and bacterial infections have been linked to increased risk of developmental delay, schizophrenia, autism, bipolar disease, and paraventricular leukomalacia (Ellman and Susser, 2009; Parboosing et al., 2013). However, to date little is known about the role of maternal malaria infection on *in utero* neurodevelopment. It is estimated that 200 million children under the age of five in low and middle-resource settings are not reaching their developmental potential as a result of exposure to environmental factors that negatively impact early neurocognitive development, including a prominent role for malaria infection (Grantham-McGregor et al., 2007). The complement system may be a common pathway involved in the regulation of healthy placental and fetal development as well as *in utero* neurodevelopmental processes.

Most complement components and receptors are expressed by astrocytes, microglia and neurons in the CNS (O’Barr et al., 2001; Veerhuis et al., 2011) and the role of the complement system in neurodegenerative processes has been well studied (Bonifati and Kishore, 2007; Woodruff et al., 2011; Orsini et al., 2014). Complement activation has been linked to neuro-inflammation in several neurodegenerative disorders including stroke, Parkinson’s disease, and Alzheimer’s disease (Zanjani et al., 2005; Banz and Rieben, 2012; Pavlovski et al., 2012). Complement-mediated recruitment and activation of glial cells, which secrete cytokines and free radicals, can disrupt neuronal function and induce neurotoxicity (Zanjani et al., 2005; Ager et al., 2010). While any specific role of the complement system in fetal neurodevelopmental processes remains unknown, it has recently been linked with numerous early neurodevelopmental processes including neurogenesis, cell migration, and synaptic pruning (Rahpeymai et al., 2006; Chu et al., 2010; Stephan et al., 2012; Pedroni et al., 2014).

Research in our laboratory using a mouse model of PM, has observed impaired learning and memory, and increased depressive-like behavior in offspring born to malaria-infected dams. This correlated with reduced regional levels of major biogenic amines (dopamine, serotonin and norepinephrine) in the frontal cortex, temporoparietal cortex, and the striatum. The behavioral phenotype and reduction in circulating levels of neurotransmitters was rescued by genetic and pharmacological blockade of signaling via the C5a-C5aR pathway (McDonald et al., 2015). Importantly, these results were observed independent of a birth phenotype (LBW or PTB). In the absence of infection-induced LBW, the inflammatory pathways induced by maternal infection can result in a fetal inflammatory response, altered angiogenesis, and changes in synaptic development that can impact *in utero* and postnatal neurodevelopmental processes (**Figure 2**). While the specific role that the complement system plays in fetal neurodevelopment continues to be elucidated, this research has important implications for malaria as many exposed pregnancies do not result in a birth phenotype but may still be at risk of adverse neurodevelopmental outcomes.

## Conclusion

The majority of all pregnancies worldwide are at risk of malaria infection (Dellicour et al., 2010). These pregnancies occur in low resource settings where the burden of adverse birth outcomes is greatest. Based on the fundamental role the complement system plays in the regulation of immune, vascular and central nervous system development, we propose that the complement system plays a central role in the pathology, adverse birth outcomes, and potential neurocognitive impairments resulting from malaria infection in pregnancy.

## Conflict of Interest Statement

The authors declare that the research was conducted in the absence of any commercial or financial relationships that could be construed as a potential conflict of interest.
